# Practical Points

**Published:** 1895-05-04

**Authors:** 


### PRACTICAL POINTS.

Questions are invited for this column on any point in the administration of hospitals and asylums which may prove of difficulty or interest to our readers. All communications should be marked "Practical Points," addressed to the Editor of The Hospital, 428, Strand, W.C.]

*Floor Polishing.—"J. D." writes: A new wing has been added to an old infirmary, and has been floored with pine blocks, which it is desired to have polished. Can you tell me how it should be done at first? The old part of the building has an ordinary board flooring, which is scrubbed. Could this be polished in any way to make it appear more uniform with the new building?*

If the old floor-boards are not too much worn to be planed smooth they can be either paraffined or wax-polished. The new block floor should be paraffined.

